# Structure of WbdD: a bifunctional kinase and methyltransferase that regulates the chain length of the O antigen in *Escherichia coli* O9a

**DOI:** 10.1111/mmi.12014

**Published:** 2012-09-27

**Authors:** Gregor Hagelueken, Hexian Huang, Bradley R Clarke, Tomas Lebl, Chris Whitfield, James H Naismith

**Affiliations:** 1 Biomedical Sciences Research Complex, University of St AndrewsNorth Haugh, St Andrews, Fife, KY16 9ST, UK; 2 Department of Molecular and Cellular Biology, University of GuelphGuelph, Ontario, N1G 2W1 Canada; 3 School of Chemistry, University of St AndrewsNorth Haugh, St Andrews, Fife KY16 9ST, UK

## Abstract

The *Escherichia coli* serotype O9a O-antigen polysaccharide (O-PS) is a model for glycan biosynthesis and export by the ATP-binding cassette transporter-dependent pathway. The polymannose O9a O-PS is synthesized as a polyprenol-linked glycan by mannosyltransferase enzymes located at the cytoplasmic membrane. The chain length of the O9a O-PS is tightly regulated by the WbdD enzyme. WbdD first phosphorylates the terminal non-reducing mannose of the O-PS and then methylates the phosphate, stopping polymerization. The 2.2 Å resolution structure of WbdD reveals a bacterial methyltransferase domain joined to a eukaryotic kinase domain. The kinase domain is again fused to an extended C-terminal coiled-coil domain reminiscent of eukaryotic DMPK (Myotonic Dystrophy Protein Kinase) family kinases such as Rho-associated protein kinase (ROCK). WbdD phosphorylates 2-α-d-mannosyl-d-mannose (2α-MB), a short mimic of the O9a polymer. Mutagenesis identifies those residues important in catalysis and substrate recognition and the *in vivo* phenotypes of these mutants are used to dissect the termination reaction. We have determined the structures of co-complexes of WbdD with two known eukaryotic protein kinase inhibitors. Although these are potent inhibitors *in vitro*, they do not show any *in vivo* activity. The structures reveal new insight into O-PS chain-length regulation in this important model system.

## Introduction

Most Gram-negative bacteria contain lipopolysaccharide (LPS) as a major component of the outer membrane. LPS consists of a well-conserved anchor, lipid A, linked to a hypervariable strain-specific O-antigen polysaccharide (O-PS) (over 180 in *Escherichia coli*) (Stenutz *et al*., 2006) via a short core oligosaccharide (Raetz and Whitfield, 2002). LPS mediates critical interactions with the host immune defences and the presence of O-PS plays a role in resistance to complement-mediated killing (Joiner, 1988).

**Fig. 1:**
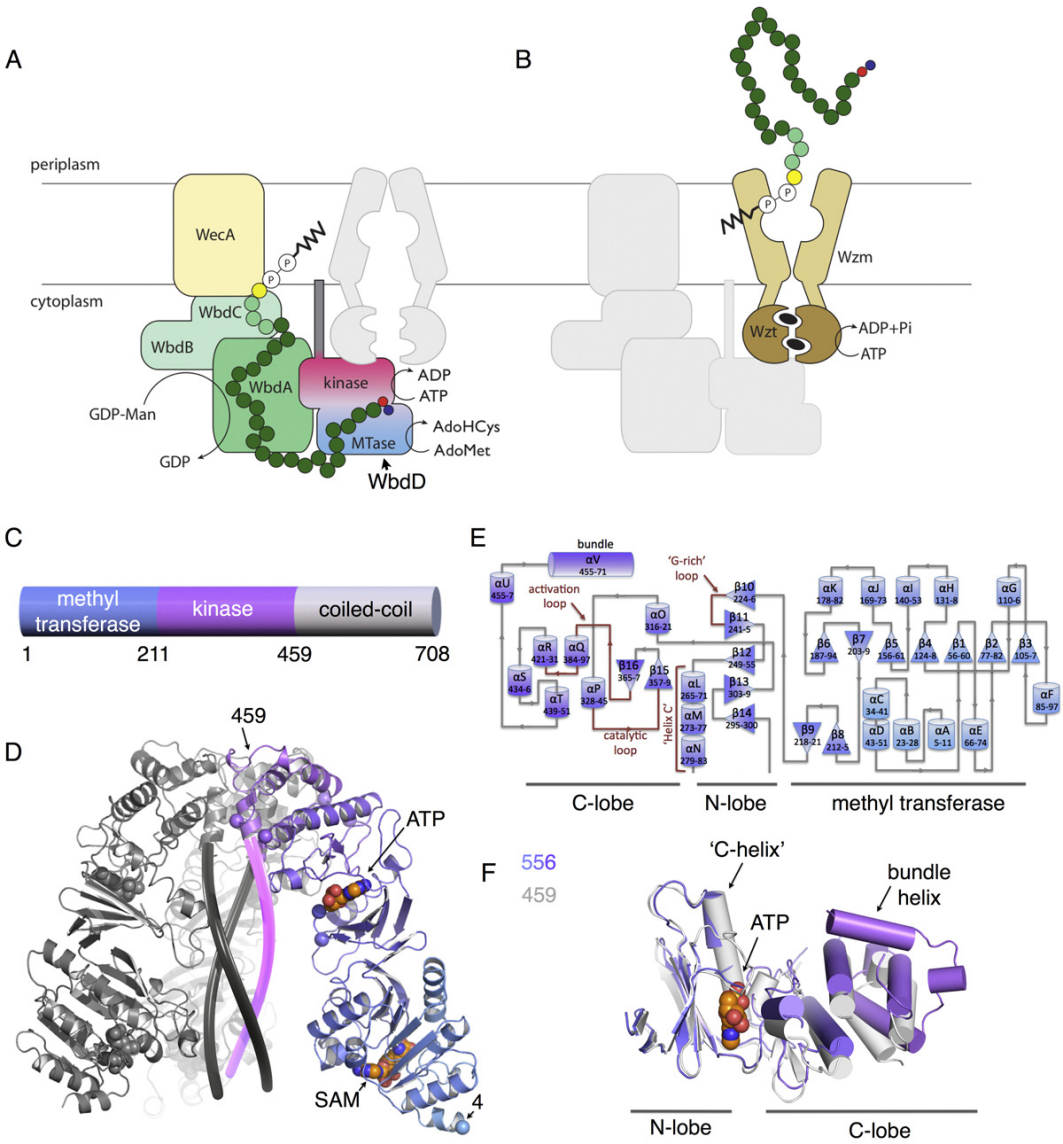
Function and structure of WbdD. A and B. (A) Overview of the biosynthesis and export (B) of the O9a O antigen. The polymannose glycan is synthesized by three mannosyltransferases with different roles in the overall process (WbdCBA). Together, WbdCB add three Man residues to the undecaprenyl-PP-GlcNAc acceptor formed by the initiating WecA enzyme. The resulting lipid-linked oligosaccharide is then extended by the polymerizing WbdA enzyme to build the repeat-unit polysaccharide. Chain extension is terminated by the dual kinase/methyltransferase activity of WbdD. Terminated polysaccharides are recognized by the carbohydrate-binding module linked to the nucleotide-binding domain (Wzt) of the ABC transporter and are exported to the periplasmic face of the inner membrane, presumably via a channel formed by the transmembrane polypeptide (Wzm). The details of the transport process have not been resolved. Once exported, the nascent O antigen is transferred from its undecaprenyl-PP carrier to the lipid A-core part of LPS (which is synthesized and exported separately) and the completed molecule is translocated to the cell surface via proteins in the Lpt pathway. C. Domain boundaries of WbdD. D. The trimeric structure of WbdD556, shown as a cartoon model with a colour gradient running form blue (N-terminus) to purple (C-terminus). The cofactors are depicted as spheres (atom colouring: C-orange, N-blue, O-red, P-light orange). The proposed structure of the C-terminal three-helix bundle is indicated by coloured tubes. E. Topology diagram of WbdD556. Strands are represented by triangles, helices by tubes. The colour scheme is identical to (D). F. Superposition of the kinase domains of WbdD556 (coloured as in Fig. 2) and WbdD459 (grey). The ATP cofactor is shown as spheres.

Although the structures of O-PSs are extremely diverse, they are made by one of three types of O-PS biosynthesis systems; the ‘Wzx/Wzy dependent’, the ‘ATP-binding cassette (ABC) transporter dependent’ and the poorly studied ‘synthase dependent’ pathways (Raetz and Whitfield, 2002). In each case, the length of the O-PS is tightly controlled and exhibits a defined range of chain lengths (termed ‘modal’ distribution) (Fig. 1A). The polymannose O-PSs of *E. coli* serotypes O8, O9 or O9a are prototypes for the ‘ATP transporter dependent’ pathway of O-PS synthesis (Fig. 1B). In this system, the WecA enzyme synthesizes a ‘primer’ comprising undecaprenol-diphospo-GlcNAc (und-PP-GlcNAc) and the polymannose O-PS is synthesized and extended by the mannosyltransferases WbdA, WbdB and WbdC (Greenfield *et al*., 2012). The length of the polymannose chain in *E. coli* O9 (and O9a) is controlled by a termination reaction, whereby a phosphate followed by a methyl group is added to the 3-OH position of the non-reducing terminal mannose residue of the chain (Clarke *et al*., 2004; 2011) (Fig. 1A). In contrast, in *E. coli* O8 a methyl group is added to the 2-OH of the reducing mannose without phosphorylation (Vinogradov *et al*., 2002; Clarke *et al*., 2004). These modifications block further polymerization and are retained in the mature O-PS structure (Vinogradov *et al*., 2002; Kubler-Kielb *et al*., 2012). The terminating and chain length regulating modifications are catalysed by the strain-specific WbdD proteins. The O9/O9a WbdD homologue is a bifunctional kinase-methyltransferase, whereas the O8 WbdD is a simple methyltransferase (Clarke *et al*., 2004; 2011). The WbdD protein has been shown to recruit the critical WbdA mannosyltransferase to the membrane via protein:protein interactions mediated by the C-terminal (non-catalytic) region of WbdD (Clarke *et al*., 2009). Chain termination by WbdD is essential for export; *E. coli* O9a *wbdD* mutants can synthesize the unmodified O9a polysaccharide but are unable to export it (Cuthbertson *et al*., 2005; 2007). This phenotype reflects a quality-control process (Cuthbertson *et al*., 2007; 2010) ensuring that only O-PS glycans of defined chain lengths are exported and WbdD is the master-regulator in the system. Comparable assembly systems are exploited in the biosynthesis of other O-PSs in various bacteria and in assembly of O-linked glycans in Gram-positive bacteria (Cuthbertson *et al*., 2010).

**Fig. 2:**
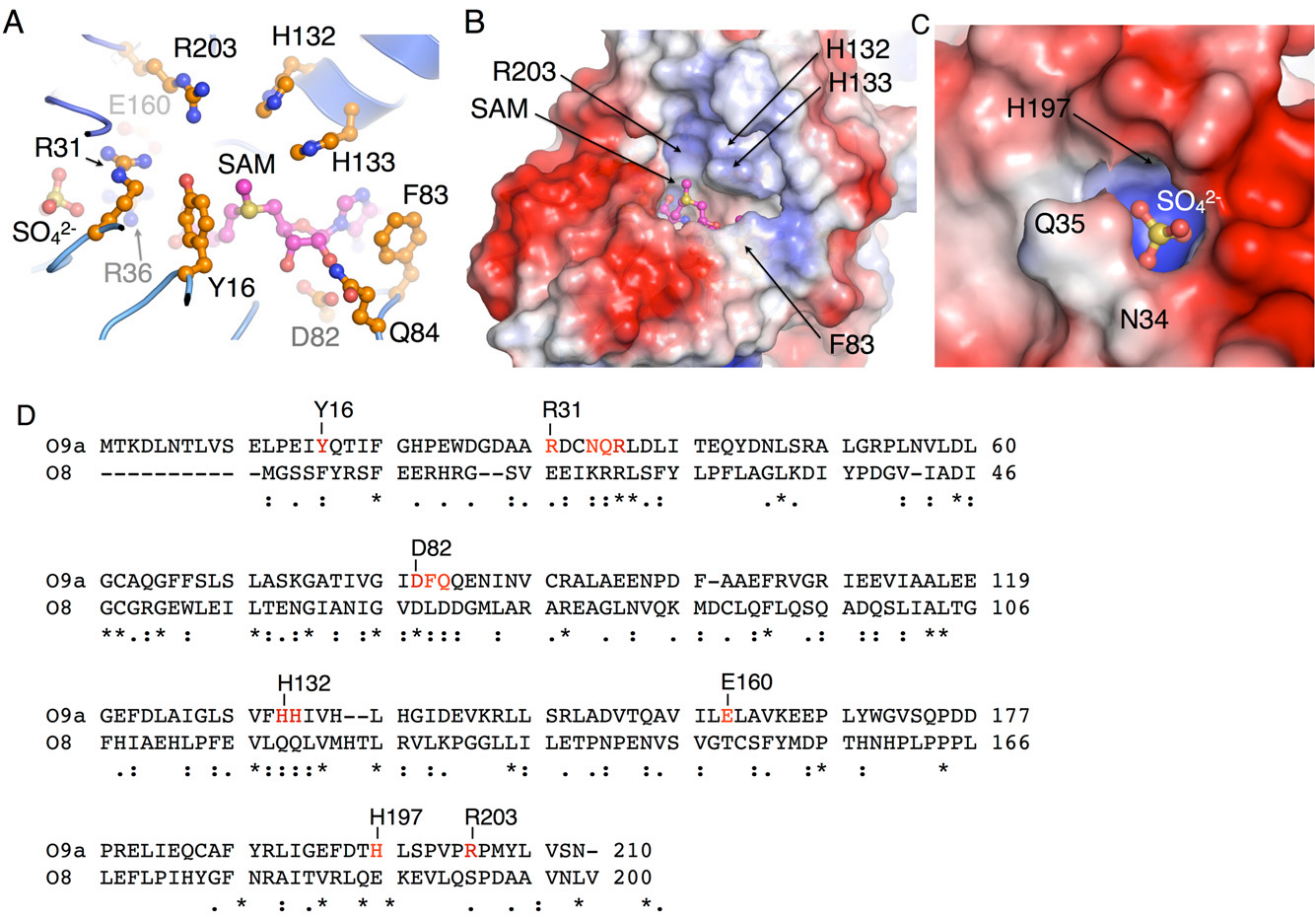
The MTase domain of WbdD556. A. Close up of the active site. The SAM cofactor (purple) and selected active-site residues are shown as ball-and-sticks models. B. Electrostatic surface potential of the MTase active site (blue-positive, white-neutral, red-negative). The SAM cofactor (purple ball-and-sticks) and selected active-site residues are numbered. C. The sulphate binding site is close to the MTase active site (colour scheme as Fig. 3B). D. Sequence alignment (clustalw) of the WbdD enzymes from *E. coli* O9a and O8 (no kinase domain). Residues that are shown in (A), (B) and (C) are indicated and highlighted in red.

Here, we report the 2.2 Å crystal structure of the bi-functional catalytic domain of the WbdD protein from *E. coli* O9a. Our data show the two domains are arranged to produce the unique methyl-phosphate modification on the 3-OH of the non-reducing terminal mannose. Most surprisingly, the kinase domain adopts a fold thought confined to eukaryotic tyrosine kinase. The structural information allows us to selectively disable the individual domains by site-directed mutagenesis. We have investigated the resulting phenotypes *in vivo* in addition to *in vitro* enzymatic activity. We report co-crystal structures and *in vitro* potency for inhibitors of the kinase domain of WbdD. The data gives important new insights into the mechanism by which the modal chain-length distribution is achieved.

## Results

### Structure of WbdD556

The 82 kDa WbdD protein from *E. coli* O9a (Fig. 1A and C) comprises 708 residues and contains three domains, an N-terminal methyltransferase (MTase) domain, a kinase domain and a C-terminal domain (residues 460–708) that includes predicted coiled-coil motifs (Clarke *et al*., 2004) (Fig. 1C). The full-length WbdD protein was not tractable for biophysical characterization but a 63 kDa fragment, comprising residues 1–556 (WbdD556), could be expressed, purified and crystallized (Hagelueken et al., 2012). Crystal optimization by dehydration and structure solution are detailed elsewhere (Hagelueken *et al*., 2012). The 2.2 Å structure was refined using REFMAC5 (Murshudov *et al*., 1997) (Fig. 1D). The R_free_ and R-factors converged at 17.9%/22.2% and the geometry of the model was checked and optimized using MOLPROBITY (Chen *et al*., 2010). Crystallographic data are summarized in Table 1.

**Table 1:** Data collection and refinement statistics

	WbdD556	WbdD556	WbdD556	WbdD459
	AMPPNP/SAM	LY294002/SAM	GW435821x/SAM	AMPPNP/SAM/D-Mannose
	PDB:4AZS	PDB:4AZT	PDB:4AZV	PDB:4AZW
Data collection				
Space group	I23	I23	I23	P2_1_2_1_2_1_
Unit cell (Å, °)	a = b = c = 159.2, α = β = γ = 90	a = b = c = 159.0, α = β = γ = 90	a = b = c = 159.5, α = β = γ = 90	a = 40.7, b = 89.6, c = 135.5, 3 = β = γ = 90
Matthews coefficient (Å^3^*Da^−1^)	2.8	2.8	2.8	2.5
Solvent content (%)	55.8	55.6	56	50.3
Molecules per ASU	1	1	1	1
Wavelength (Å)	0.9686	1.5417	0.9795	0.9173
Beamline	I04	Inhouse	I04	I04-1
Resolution range (Å)	65.0 − (2.21 − 2.15)	42.5 − (2.40 − 2.34)	112.8 − (3.32 − 3.24)	67.7 − (2.53 − 2.47)
Total observations	402776	246972	63966	119284
Unique reflections	36526	27997	10923	18171
Completeness (%)	100 (100)	99.3 (91.3)	99.9 (100)	98.5 (95.0)
R_merge_	0.09 (0.1)	0.06 (0.41)	0.12 (0.70)	0.08 (0.75)
Multiplicity	11 (11.2)	8.8 (2.8)	5.9 (6.0)	6.6 (6.1)
I/σ/I	19.3 (3.2)	27.1 (2.4)	11.5 (2.6)	19.3 (3.1)
Refinement				
Resolution range (Å)	65.0–2.15	112.4–2.34	112.8–3.29	67.7–2.47
R/R_free_ (%)	17.9/22.2	18.8/25.5	22.3/26.5	19.3/23.7
R.m.s.d. bonds (Å)/angles (°)	0.013/1.68	0.014/1.78	0.008/1.36	0.014/1.67
Ramachandran plot (% favoured/disallowed)	97.2/0.2	95.6/0.9	97.3/0.2	97.3/0.3
MOLPROBITY (Chen *et al*., 2010) score/clash score	1.91/8.33	2.38/14.41	2.01/10.79	2.09/10.19

Values in parenthesis refer to the highest resolution shell.

The WbdD556 structure has an elongated shape with an N-terminal MTase domain (residues 1–210) directly adjacent to the kinase domain (residues 211–459) (Fig. 1C–E). WbdD556 is a trimer in the crystal, consistent with its status in solution as judged by gel-filtration (Fig. S1). The C-terminal α-helix (αV) (residues 459–473) of each monomer wraps around the crystallographic threefold axis forming a trimeric bundle reminiscent of a coiled-coil (Fig. 1D). Residues 474–556 are disordered, possibly because of packing constraints (Hagelueken *et al*., 2012) but are predicted to be helical. Removing this C-terminal helix (WbdD459) gave a monomeric protein in crystals (space group P2_1_2_1_2_1_, Table 1) and in solution (Fig. S1), confirming that the helical bundle drives trimer formation. The structures of WbdD459 and monomers of WbdD556 are very similar [r.m.s.d. = 1.5 Å (2538 superimposing atoms)], but there is a rigid-body rotation around the hinge connecting the N- and C-lobe within the kinase domain, accounting for the relatively large r.m.s.d. (Fig. 1F).

### The methyltransferase domain is specific to a phosphate attached to 2-α-d-mannosyl-d-mannose

The MTase domain of WbdD consists of a seven-stranded β-sheet with the characteristic (for MTases) reversed β-hairpin (β6-β7) at the C-terminus of the sheet, which is surrounded by α-helices on either side (Fig. 1E). The fold belongs to group 1 according to a classification system for methyltransferases proposed by Schubert *et al*. (2003). NodS from *Bradyrhizobium japonicum* (Cakici *et al*., 2010) and RebM of *Lechevalieria aerocolonigenes* (Singh *et al*., 2008) are the most similar structures (NodS: Z-score 16.0, r.m.s.d. 2.7 Å; RebM: Z-score 15.6, r.m.s.d. 2.8 Å). The *S*-adenosylmethionine (SAM) binding site located on top of the β-sheet (Fig. 2) (Fig. S2). Positively-charged residues (R203, H132 and H133) cluster around the electrophilic methyl group, consistent with a phosphate binding site (arising from the phosphorylated sugar substrate) (Fig. 1A–C). R203 is not structurally conserved in NodS or RebM and the two His residues are found as either two His or two Tyr in these two proteins (Fig. S2).

**Fig. 3:**
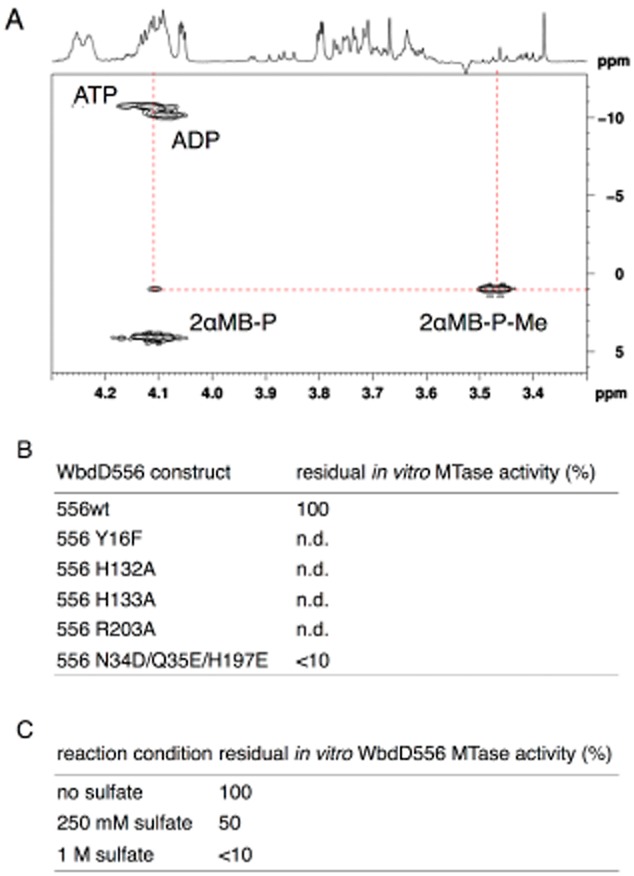
*In vitro* and *in vivo* analysis of the MTase activity of WbdD. A. Monitoring activity of WbdD by ^1^H,^31^P-HMBC correlation. The cross-peak δ_H_ = 4.14 ppm δ_P_ = 4.1 ppm indicates formation of phosphorylated 2α-MB. A new phosphorus resonance at 1.0 ppm appears after adding SAM. Two corresponding cross-peaks (δ_H_ = 3.47 and 4.11 ppm) are in accordance with 2α-MB methyl phosphate having two non-equivalent hydrogen atoms in distance of three bonds from phosphorus. B. Table of NMR results for different mutants. Figure S6 shows that the MTase mutants are still capable of phosphorylating 2α-MB. C. The influence of sulphate on the reaction velocity *in vitro*.

The MTase reaction was analysed by NMR using ^1^H,^31^P-HMBC correlation spectra and the kinase substrate 2α-mannobiose (2α-MB) (Fig. 3A). A cross-peak at δ_H_ = 4.14 ppm δ_P_ = 4.1 ppm indicated formation of phosphorylated 2α-MB. A new phosphorus resonance at δ_P_ = 1.0 ppm appeared after SAM was added to the reaction establishing methylation of the 3-phosphate group (Fig. 3A); consistent with previous studies using polymeric material (Clarke *et al*., 2011; Kubler-Kielb *et al*., 2012). No such cross-peak appears when d-mannose-3-phosphate (monomer), d-mannose-1-phosphate, d-mannose-6-phosphate, d-glucose-1-phosphate or phosphate were added to the reaction, demonstrating the methyltransferase activity is specific to phosphate attached to the 3-position of the disaccharide. Mutant WbdD556 proteins (H132A, H133A, R203A, Y16F) were able to phosphorylate 2α-MB but no MTAse reactivity was detected *in vitro* (Fig. 3B).

Unlike other structurally characterized MTase proteins, WbdD has a pocket lined by R31, R36, N34, Q35, C33, F194, D195 and H197 adjacent to the active site. The pocket is occupied by sulphate in the WbdD556 structure, these crystals were grown from ammonium sulphate solutions (Fig. 2A and C) whereas the pocket is empty (with the loops disordered) in WbdD459 crystals which were grown from a non-sulphate-containing solution (Fig. S3). Running the identical NMR-based methylation assay with Wb556 in 250 mM sulphate reduces the reaction rate by 50% and in 1 M sulphate (similar to crystallization condition) the rate is down to < 10% (Fig. 1C). A triple N34D, Q35E and H197E mutant [constructed to disrupt the sulphate-binding pocket (Fig. 2C)] has less than 10% of the wild-type reaction rate (Fig. 3B).

### The kinase domain of WbdD resembles eukaryotic kinases

**Fig. 4:**
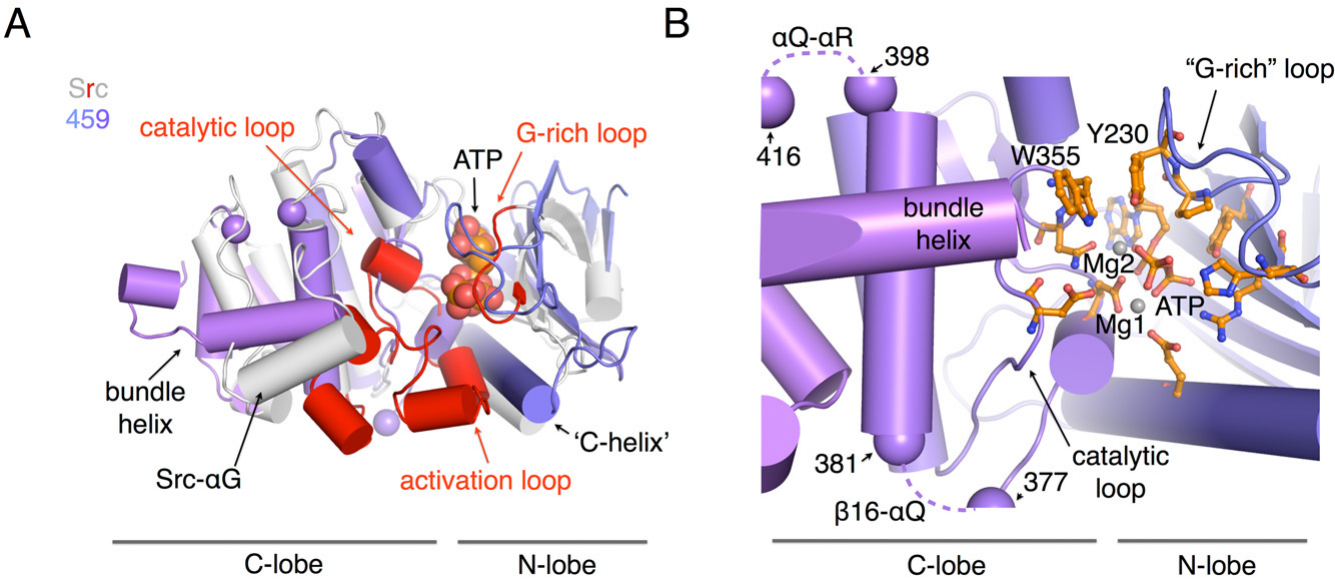
Analysis of the kinase domain of WbdD. A. Superposition of WbdD459 and human Src kinase (grey). The signature motifs of Src-kinase such as the catalytic loop, the activation loop (A-loop) and the Gly-rich loop are highlighted in red, the position of the C-helix is indicated. Helices are pictured as cylinders and the colour scheme is as in Fig. 1. B. Selected active-site residues of WbdD (the WbdD459 structure is shown) are shown as sticks. The start- and end-points of disordered loops (activation loop β16-αQ and αQ-αR) are marked by spheres and the loops are represented as dashed lines.

In the kinase domain of WbdD, the N- and C-lobes are separated by an ATP binding cleft (Fig. 1C–F). The DALI server (Holm and Rosenström, 2010) identifies a strong structural similarity to well-studied eukaryotic kinases including the Src kinase [PDB-id: 2SRC, Z-score: 12.2, r.m.s.d.: 3.3 Å, length of alignment: 182 residues (Xu *et al*., 1997)], ROCK (Jacobs *et al*., 2006), Lck (Yamaguchi and Hendrickson, 1996) and Erk (Canagarajah *et al*., 1997) (Fig. 4A). The overall sequence identity between the kinase domains of WbdD and human Src is very low, with 11% over 295 aligned residues. Interestingly, Src (PDB: 2SRC) superimposes more closely on WbdD459 (r.m.s. = 3.3 Å (350 Cα atoms) than on WbdD556 [r.m.s. = 5.6 Å (350 Cα atoms)]. Although the characteristic structure of the catalytic loop is conserved in WbdD (Fig. 4A), superposition of Src and WbdD459 reveals that two other key features of eukaryotic kinases (Endicott *et al*., 2012), the ‘G-rich’ loop (β10–β11 in WbdD) and the activation loop (β16–αQ in WbdD) are different in WbdD (Fig. 4A). Of our structures, the ‘G-rich’ loop is most clearly defined in the Wbd459 structure, where it fulfils the same role as in Src; co-ordination of the AMPPNP (ATP mimic). However, in WbdD the loop protrudes further into the ATP binding cleft than the corresponding element in Src (Fig. 4A) and it interacts with the catalytic loop through a π-stacking between W355 and Y230 (Fig. 4B). The ‘activation loop’ of Src contains a regulatory tyrosine residue that can be autophosphorylated to activate the kinase. In WbdD, the corresponding loop (β16–αQ) is much shorter, lacks tyrosine and is partly disordered in all of our structures (Fig. 4A and B). The C-terminal helix αV of WbdD (three-helix bundle) is a structural permutation of the eukaryotic kinase fold and occupies a similar position as the αG helix of Src (Fig. 4A) that stabilizes the activation loop in Src.

WbdD binds ATP (or AMPPNP) via two Mg^2+^ ions (denoted Mg1, Mg2) that are co-ordinated by N356, D369 [part of the DFG motif which is almost universally conserved in kinases (Kannan *et al*., 2007; Endicott *et al*., 2012)], E274 and all three phosphate groups of ATP (or AMPPNP), as well as by two water molecules (Fig. 4B). Mg1 bridges the α- and γ-phosphates, Mg2 the β- and γ-phosphates. This co-ordination resembles that seen in cAMP-dependent protein kinases (Zheng *et al*., 1993a; 1993b)

### Substrate specificity of the kinase activity of WbdD

**Fig. 5:**
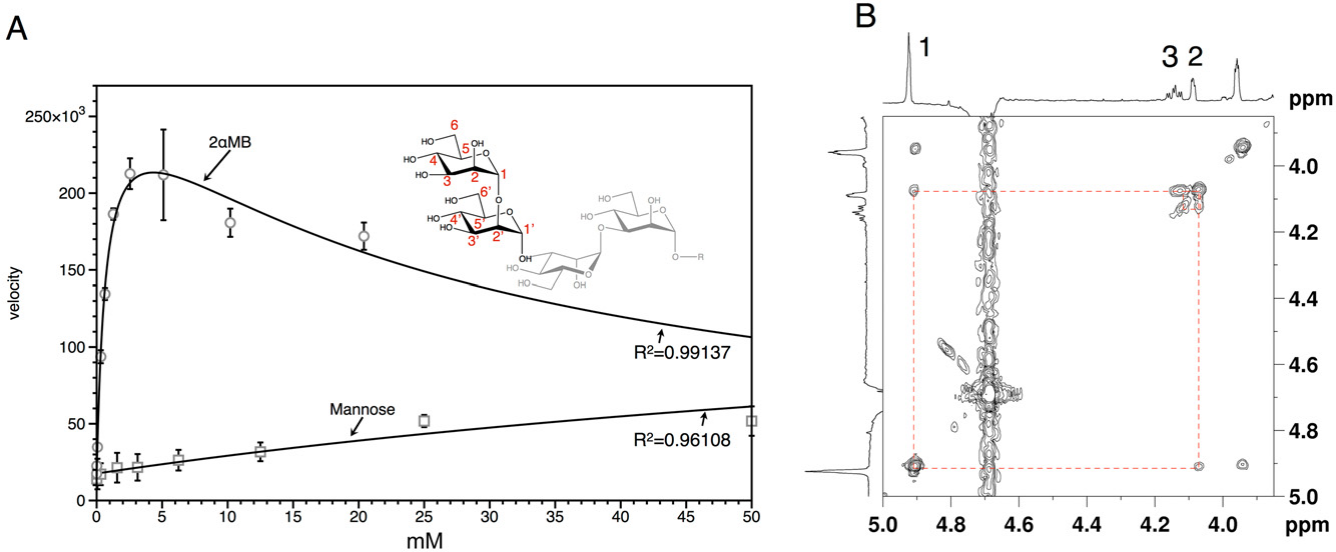
Kinase activity. A. Enzyme kinetics of the WbdD556 kinase reaction. The reaction velocity in arbitrary units (ADP glo signal, Promega) is plotted against the concentration of d-mannose and 2α-MB. Data points were measured in triplicate and error bars represent the standard deviation of the measurements. Solid lines are non-linear fits to the data points using the function *y* = offset + *V*_max_ ∗ {*x*/[*K*_m_ + *x* ∗ (1 + *x*/*K*_i_)]}. B. ^1^H,^1^H-COSY spectrum of the product yielded by phosphorylation reaction of 2α-MB with WbdD556 and ATP-D_6_. The red dashed line highlights the step-wise correlation between H1, H2 and H3 resonances of the non-reducing sugar. The resonance at 4.14 ppm (H3) shows coupling to phosphorus in ^1^H,^31^P-HMBC correlation identifying the product as 2α-MB-3-phosphate.

**Fig. 6:**
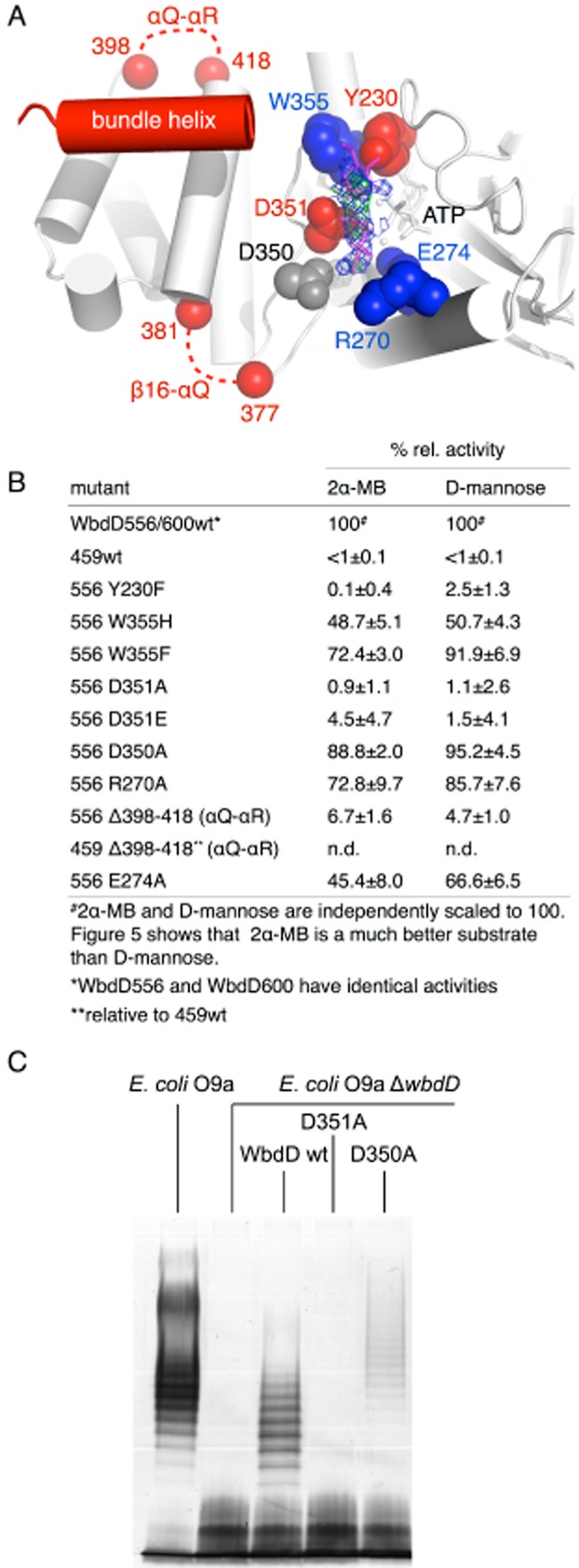
Mutagenesis of the WbdD kinase active site. A. Additional electron density that was found after the crystals were soaked with d-mannose is shown as blue mesh (2Fo-Fc, 1 σ) and green mesh (Fo-Fc, 3 σ). Two d-mannose residues are tentatively modelled into the density to show that the size of the density is consistent with two d-mannose units. WbdD is highlighted corresponding to influence of mutations at these points on the activity of the enzyme: red – < 5% WT activity, blue – ∼ 50% reduced activity, grey – 90–100% of wild-type activity. B. The relative activity of WbdD mutants for 2α-MB and d-mannose. Values are given in % and correspond to averages of triplicate measurements. The values for 2α-MB and d-mannose were independently scaled to 100. C. Silver-stained LPS SDS-PAGE showing restoration of O9a O-PS biosynthesis in an *E. coli* O9a wbdD mutant overexpressing His_6_-WbdD and two kinase active-site mutants.

WbdD556 has a marked preference for 2α‐MB over d‐Mannose with respective *K*_M_ values of ∼ 0.7 mM versus > 100 mM (Fig. 5A). Different disaccharide variants (3‐α‐, 4‐α‐ and 6‐α‐mannobiose) are also substrates, but at ∼ 80% reduced reaction rates (when compared at *K*_M_ of 2α‐MB, Fig. S4A and B). d‐glucose, d‐galactose, kojibiose (α(1→2)d‐glucosyl‐d‐glucose), lactose [β(1→2)d‐galactosyl‐d‐glucose] and maltose [α(1→4)d‐glucosyl‐d‐glucose] are not substrates (Fig. S4B). NMR analysis established that WbdD556 phosphorylates 2α‐MB at the 3‐hydroxyl of the non‐reducing sugar, identical to the authentic substrate (Clarke *et al*., 2011; Kubler‐Kielb *et al*., 2012). Similar experiments were conducted with d‐mannose, 3‐α‐, 4‐α‐ and 6‐α‐mannobiose and indicate these compounds are also phosphorylated at the 3‐hydroxyl of the non‐reducing sugar (Fig. S4C). WbdD459, which lacks the C‐terminal bundle‐helix αV (Fig. 1C–F), showed a marked decrease in kinase activity but has lost the preference for 2α‐MB over d‐mannose (Fig. S4D and E). Thus, the C‐terminal bundle structure is clearly important both for biochemical activity and for polymer (substrate) recognition or co‐ordination. We speculate that the C‐terminal bundle interacts with and stabilizes active‐site features, such as the activation loop (β16/αQ) or loop αQ/αR, which are disordered in our structures (see below, Fig. 6A).

### Mutational analysis of the kinase substrate (acceptor) binding site

Attempts to co-crystallize WbdD556 with 2α-MB were unsuccessful, potentially because of the rigid body movements occurring in the structure during dehydration (Hagelueken *et al*., 2012). We therefore attempted to co-crystallize WbdD556 and WbdD459 with d-mannose (*K*_M_ > 100 mM) by soaking our crystals in saturated d-mannose solutions (∼ 1.6 M). Only WbdD459 crystals (with AMPPNP) withstood the treatment and gave additional electron density (not present before addition of d-mannose) that we interpret as two mannose units, Fig. 6A. The relatively poor quality of this density is consistent with the anticipated weakness of the interaction. However, it allowed us to identify amino acids surrounding the active site for mutagenesis (Fig. 6A and B). The D351E/A mutations and the Y230F mutation completely eliminated activity *in vitro*. Mutation of W355 to F had very little effect on catalysis, whereas W355H showed a ∼ 50% reduction of activity. These data are consistent with a structural model where substrate is bound by the hydroxyl of Y230 and the carboxylate of D351. However, since D351 also ligates the Mg1 ion, its mutation may also disrupt catalysis. W355 does not play a catalytic role and we suggest instead that it helps to anchor the side-chain of Y230 by a π-stacking interaction (Figs 4B and 6A). The R270A and E274A mutations target residues that are distant from the non-reducing mannose unit (which is phosphorylated) but are predicted to recognize the second sugar molecule. Indeed, the mutants lead to a more pronounced loss of activity towards 2α-MB compared with mannose (Fig. 1A and B). Deletion of either the C-terminal bundle helix (WbdD459, see above), or loop αQ–αR, severely attenuates the activity of WbdD against both 2α-MB and d-mannose and completely removes any preference for the polymeric substrate (Fig. S4C and D, Fig. 1A and B).

Kinase-dead mutants such as D351A lead to a complete abrogation of O-PS synthesis *in vivo*, comparable to the Δ*wbdD* strain (Fig. 6C). D350A which showed a 10% decrease in activity *in vitro* (Fig. 6B) shows a shift to longer chain length *in vivo*, combined with a decrease in the amount of polymer that is expressed on the surface (Fig. 6C). These characteristics are the anticipated result of a mutation with reduced catalytic activity that simultaneously affects the ability to control chain length appropriately and reduces the terminated product available for recognition and export by the ABC transporter (Clarke *et al*., 2004; Cuthbertson *et al*., 2007).

### WbdD is inhibited by small-molecule inhibitors of eukaryotic kinases

**Fig. 7:**
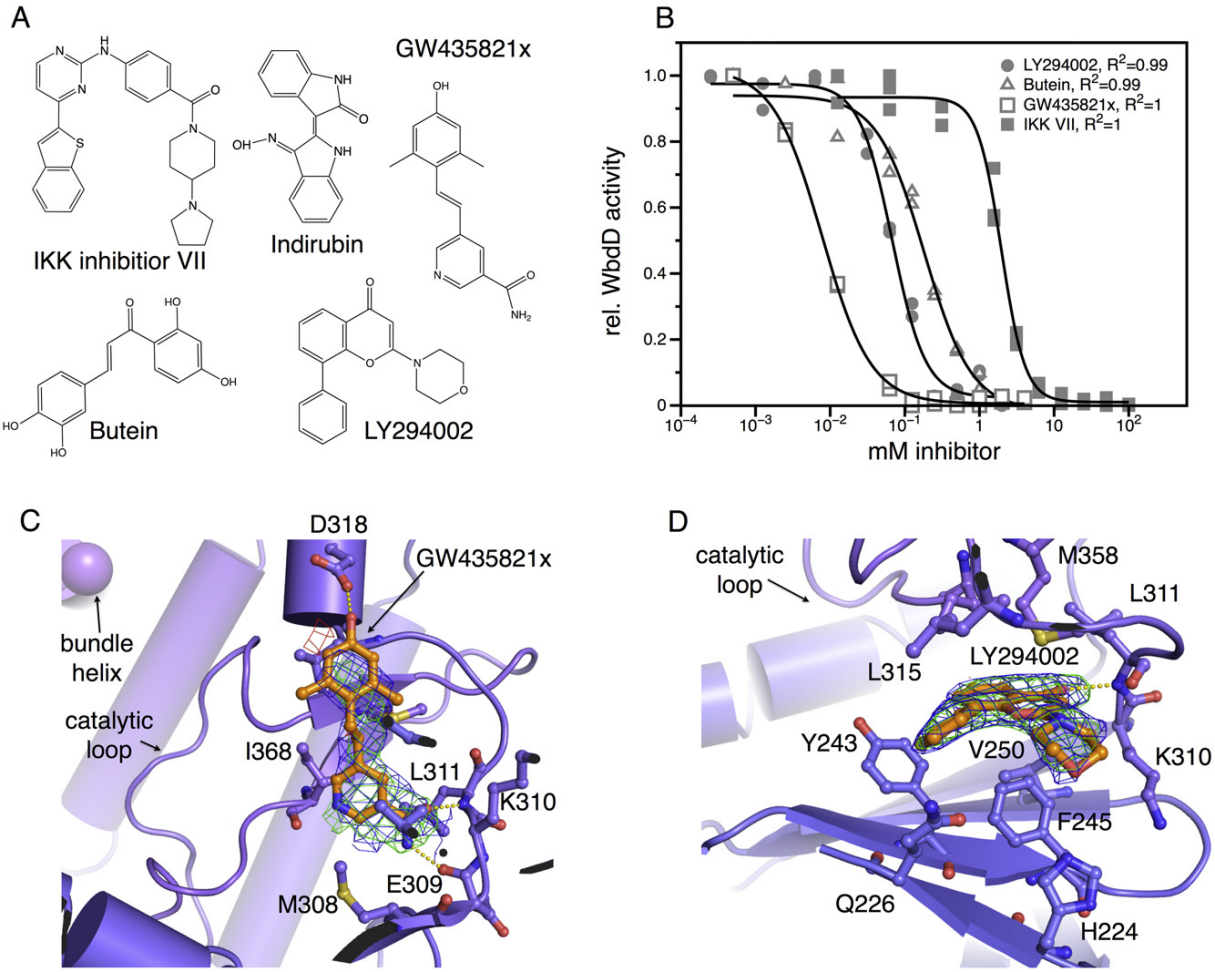
Inhibition of WbdD by tyrosine kinase inhibitors. A. Chemical structures of compounds that inhibit WbdD. B. IC_50_ analysis of the compounds shown in (A). Indirubin is not shown, since no IC_50_ data could be produced due to solubility issues. C. Co-crystal structure of WbdD with GW435821x. The inhibitor and selected residues are indicated as ball-and-stick. The blue mesh is a 2DFo-mFc omit density at 1 σ, the green mesh a DFo-mFc omit density at 3 σ. D. Co-crystal structure of WbdD with LY294002; representation as (C). Stereo pairs for (C) and (D) are in Fig. S7.

Approximately 1000 different kinase inhibitors were screened from collections obtained from GlaxoSmithKline and the International Centre for Kinase Profiling (Dundee, UK). IC_50_ values were determined for GW435821x (7.8 μM), LY294002 (68 μM), Butein (182 μM), IKK inhibitor VII (1.2 mM) and indirubin (> 100 μM; estimated due to low solubility) (Fig. 7A and B). Co-complex structures of the two best inhibitors GW435821x and LY29004 were solved at 3.3 Å and 2.3 Å resolution respectively (Fig. 7C and D, Table 1). Both compounds occupied the ATP-binding cleft and interacted with the protein via multiple hydrophobic and vdW interactions. GW435821x is also bound by three hydrogen bonds to D318, the backbone oxygen of E309, and K310. LY294002 only makes a single polar interaction to the backbone nitrogen of K310 (Fig. 7D), consistent with its lower affinity. None of the inhibitor compounds tested affected either growth or the O-PS phenotype of *E. coli* O9a [CWG634 (Clarke *et al*., 2004)], even when cells were grown in the presence of sub-inhibitory concentrations of EDTA in an attempt to permeabilize the cell envelope. Our data do not reveal the reason for the lack of activity but lack of uptake, rapid efflux or metabolism provide some potential explanations.

## Discussion

Regulation of carbohydrate polymerization and export is vital to bacterial pathogenesis. The *E. coli* O9a system is an important prototype for these processes in ABC transporter-dependent glycan biosynthesis (Cuthbertson *et al*., 2010). Phosphorylation by WbdD is sufficient to terminate the glycan chains (Clarke *et al*., 2011) and we have shown here that WbdD mutations which reduce the kinase activity *in vitro* lead to much longer O-PS chain lengths *in vivo* (e.g. WbdD D350A, Fig. 6C). In the absence of all phosphorylation, no O-PS is seen on the cell surface (WbdD D351A, Fig. 6C) because the terminal modification is essential for recognition of the nascent glycan by the carbohydrate-binding module attached to the ABC transporter (Cuthbertson *et al*., 2007).

WbdD is an unusual enzyme since it combines both a phosphate-methyltransferase and a sugar kinase activity. Whereas the more common 6- or 1-mannose-phosphates are produced by mannokinase and phosphomannomutase respectively (Sebastian and Asensio, 1972; Sugiyama *et al*., 1994), WbdD is a unique and highly specific eukaryotic-like kinase targeting the 3-OH position of d-mannose and d-mannose-based polysaccharides, with a particular preference for α(1→2) linked mannose units. Site-specific phosphorylation of sugar polymers are difficult to achieve chemically and our results show that WbdD can tolerate a number of different mannose-containing disaccharides while regio-selectively phosphorylating the 3-OH. Although existence of eukaryotic-like kinases in prokaryotes is known (Kannan *et al*., 2007), structural information is still sparse (Table S1). Examples include the aminoglycoside kinase APH from *Enterococcus faecalis* (Hon *et al*., 1997), the stress response regulator Ser/Thr kinase YihE from *E. coli* (Zheng *et al*., 2007), and the transmembrane signalling Ser/Thr kinase PknB from *Mycobacterium tuberculosis* (Young *et al*., 2003). Among bacteria, close homologues of the WbdD kinase domain are found in some *E. coli* strains and related enterobacteria (*Klebsiella, Salmonella, Sodalis, Pantoea*), as well as several *Xanthomonas* species. Although WbdD is structurally similar to the Src kinase (Fig. 4A) it does not easily fit into a sequence-based classification of kinases (Kannan *et al*., 2007), rather it shares some similarities with the PknB family, and with KdoK [3-deoxy-d-manno-octulosonic acid (Kdo) kinase] (White *et al*., 1999). The degree of evolutionary divergence of WbdD even from these kinases is obviously rather large, hindering attempts to draw firm conclusions about its evolutionary origin. The structural similarity shared with human kinases is emphasized by our identification of several known tyrosine kinase inhibitors that also inhibit WbdD. As expected the inhibitors bind in the ATP binding cleft.

The domain architecture of WbdD (eukaryotic-kinase followed by a long coiled-coil and a C-terminal protein–protein interaction domain) (Clarke *et al*., 2004; 2009) is strikingly similar to DMPK (Myotonic Dystrophy Protein Kinase)-family kinases such as ROCK (Rho-associated protein kinase). ROCKs are large (160 kDa), dimeric multifunctional protein serine/threonine kinases that regulate eukaryotic cell motility. ROCKs comprise a short N-terminal dimerization domain, a kinase domain, a long (∼ 680 AA) coiled-coil domain followed by a Rho-binding (RB) domain and a pleckstrin homology (PH) domain (Riento and Ridley, 2003). The crystal structures of ROCK I and a proteolytic fragment of its coiled-coil domain have been solved in isolation (Jacobs *et al*., 2006; Tu *et al*., 2011), so that it is not yet clear how the structural elements are connected to each other (Tu *et al*., 2011). The commonality in structure however could suggest that WbdD might be an important evolutionary link between ROCK kinases and their relatives of bacterial origin (Fig. S5).

The MTase reaction is very unusual and we have shown it to be specific for di-mannose 3-phosphate-containing acceptors. To our knowledge, the MTase domain of WbdD is the first phosphate MTase to be structurally characterized and it provides potential opportunities in biotransformation. The WbdD homologue from *E. coli* O8 lacks a kinase domain and simply methylates sugar hydroxyls directly and, as a consequence, the sequence similarity between the proteins is relatively limited. WbdD residues Arg36 and Asp82 that interact with the SAM donor are conserved in both proteins (Fig. 2A and D) but the positively charged residues Arg203, His132 and His133, which are all essential for the MTase activity of the O9a enzyme, have no equivalent in the O8 enzyme (or in other known sugar MTases) (Fig. 2A, B and D, Fig. S2). It is these residues that we identify as conferring the ability to process a phosphate nucleophile. It remains unclear why *E. coli* O9a employs a bi-functional WbdD enzyme when the simpler methyltransferase-only homologue suffices for *E. coli* O8.

The O9a system provides an elegant solution to the requirement for quality control in the chain length of bacterial glycans. The C-terminal coiled-coil domain of WbdD is essential for the recognition of correct polymeric substrate (over simple monosaccharide), important for kinase activity and required for trimer formation. Since the C-terminal region of the protein also recruits the WbdA mannosyltransferase (Clarke *et al*., 2009), we suggest that the C-terminal helix underpins the regulation of the polysaccharide chain length. The binding of substrate or product by WbdD could for example induce a conformational change, which is transmitted by the C-terminal helix and acts a ‘stop-signal’ for the polymerase. In addition or as an alternative the spatial arrangement of the catalytic domains of WbdD relative to active site of site of WbdA polymerase acts as a molecular ruler, which caps polymers of a defined length.

## Experimental procedures

### Phenotypic analysis of *E. coli* O9a harbouring wbdD site-directed mutations

CWG900 (Δ*wbdD*) (Clarke *et al*., 2009) harbouring pBAD24-based plasmids expressing His_6_-tagged mutant derivatives of *wbdD* were grown for 16 h at 37°C in LB broth containing ampicillin (100 μg ml^−1^) and d-glucose (0.4% w/v). Five ml volumes of LB broth containing ampicillin (100 μg ml^−1^) and d-mannose (0.1% w/v) were inoculated 1:50 from the starter cultures and incubated at 37°C until the OD_600_ achieved 0.4–0.6. For SDS-PAGE, LPS was prepared from 1 OD_600_ unit of cells by proteinase K treatment of whole-cell lysates (Hitchcock and Brown, 1983). SDS-PAGE was performed in Tris-glycine buffer (Laemmli, 1970) and LPS was visualized by silver staining (Tsai and Frasch, 1982).

### Cloning and protein expression

The procedures used for the cloning, expression and purification of WbdD556 have been described previously (Hagelueken *et al*., 2012). The WbdD459 construct was derived from Wbd556 by introducing a stop codon at position 459 using site-directed mutagenesis (Liu and Naismith, 2008). The expression and purification of WbdD459 were identical to the procedures used for WbdD556 (Hagelueken *et al*., 2012). Mutants of WbdD459 and WbdD556 were produced by site-directed mutagenesis (Liu and Naismith, 2008). Expression and purification procedures for the mutant proteins were again identical to the wild-type constructs.

### Crystallization, structure solution and refinement

WbdD556 was crystallized as previously reported (Hagelueken *et al*., 2012). Initial crystallization trials for WbdD459 were performed using a Honeybee 963 robot system (Genomic Solutions), using both commercially available and self-made (Oke *et al*., 2010) crystallization screens. For each of the 96-well sitting-drop vapour-diffusion screens (MRC plates, Greiner), 150 nl of protein solution (100 mg ml^−1^, including 5 mM of each AMPPNP, SAM and MgCl_2_) was mixed with 150 nl precipitant and equilibrated against a reservoir of 75 μl of precipitant. The sealed plates were then incubated at 293 K. Initial WbdD459 crystals were found in condition 93 of the JCSG+ screen (Newman *et al*., 2005). This condition was then optimized and best crystals (judged by eye) were obtained from a mixture of 20.5% PEG 3350, 0.15 M magnesium chloride, 0.10 M Bis-Tris pH 5.5 and 5 mM of each AMPPNP and SAM. Crystals (∼ 50–100 μm) usually appeared after 4–5 days at 293 K. Co-crystals of WbdD556 with kinase inhibitors were produced by soaking. The compound was dissolved in DMSO (100 mM) and added to the mother liquor of existing crystals. The plate was re-sealed and incubated at room temperature overnight. Soaked crystals were then treated with our previously described dehydration and flash cooling procedure (Hagelueken *et al*., 2012) before diffraction data were collected. Co-crystals of WbdD459 with d-Mannose were produced by transferring the WbdD459 crystals to a saturated Mannose solution prior to flash cooling in liquid nitrogen for data collection.

Diffraction data sets were collected using an in-house generator (Rigaku MicroMax™-007 HFM) and synchrotron beamlines I04 and I04-1 at Diamond (Didcot, UK). Data sets were processed using XIA2 (Winter, 2010) and structures were solved by molecular replacement using the separate domains of the low resolution WbdD556 structure as search models for PHASER (McCoy *et al*., 2007). Structures were refined with REFMAC5 (Murshudov *et al*., 1997), PHENIX.REFINE (Adams *et al*., 2002) and COOT (Emsley and Cowtan, 2004). The stereochemistry of the models was evaluated with MOLPROBITY (Chen *et al*., 2010). Parameters of data collection, refinement and stereo chemical parameters are listed in Table 1.

Data deposition: The atomic co-ordinates and structure factors reported in this article have been deposited in Protein Data Bank, http://www.pdb.org [PDB IDs: 4azs – 2.2A WbdD556 structure; 4azt – WbdD556/LY294002; 4azv – WbdD556/GW435821x; 4azw – WbdD459].

### ADP production assay

The ADP-Glo kinase Assay Kit (Promega) was used to analyse the kinetics of WbdD556 and WbdD459 with different substrates, to compare the reactivity of mutant proteins and to produce IC_50_ data. The reaction mix was prepared in a total volume of 15 μl and contained 1 μM ATP, 1 μM enzyme and substrate (e.g. 2 mM 2α-MB or 100 mM d-Mannose, see Fig. 6 for concentrations that were used for enzyme kinetics) in 1× kinase buffer (100 mM, Bis-Tris, pH 7.9, 100 mM NaCl and 5 mM MgCl_2_). The mix was incubated inside an opaque white 96-well plate (Greiner) at room temperature (RT) for 1.5 h with shaking. In the second step, 15 μl of ADP-Glo™ Reagent was added to each well and the plate was incubated for another 40 min at RT with shaking. In the last step, 30 μl of Kinase Detection Reagent was added to each well and the plate was again incubated for 1 h at RT. The luminescence was then recorded using a plate reader (Infinite® 200 PRO NanoQuant, TECAN GROUP) and the data were analysed with MS Excel and DataGraph (Visual Data Tools). To determine the IC_50_ values of kinase inhibitors, the compounds (dissolved in DMSO) were added in a range of concentrations (from 0.5 μM to 100 mM, Fig. 7) in the first step of the ADP-Glo^TM^ experiment. All experiments were performed in at least triplicate.

### NMR

To analyse the kinase reaction of WbdD556 by NMR, 50 μl of substrate (e.g. 2α-MB at 50 mM), 7 μl of ATP (100 mM), 3.5 μl of MgCl_2_ (2 M) and 70 μl of D_2_O were adjusted to 700 μl with kinase reaction buffer (100 mM Tris-HCl pH 7.9, 100 mM NaCl, 5 mM MgCl_2_). The mixture was then transferred to a standard NMR tube and 100 μg of WbdD556 in 50 μl of kinase reaction buffer were added to the tube. The contents of the tube was mixed by inverting the tube and the reaction was followed at 37°C for several hours. To avoid ATP peaks in the ^1^H spectra, deuterated ATP (ATP-D_6_, SIGMA) was used in some experiments. NMR spectra were acquired using a Bruker AVANCE 500 instrument equipped with tuneable broadband inverse (BBI) probe. To follow the MTase reaction, the kinase reaction was set up in an NMR tube as above and left to react overnight at 37°C. The ^1^H spectrum was recorded to verify the completion of the kinase reaction before 70 μl of 100 mM SAM (SIGMA) and 100 μg of fresh WbdD556 (wild type or mutants) in 50 μl of kinase reaction buffer were added. To analyse the effect of sulphate on the reaction, 5.7 M ammonium sulphate was added to a final concentration of 250 or 1.1 M sulphate. The MTase reaction was followed at 37°C.
