# Supplementary material for: Structure of WbdD: a bifunctional kinase and methyltransferase that regulates the chain length of the O antigen in Escherichia coli O9a
Source: Mol Microbiol. 2012 Sep 27;86(3):730–42. doi: 10.1111/mmi.12014 (PMC3482155; doi:10.1111/mmi.12014)
Supplement: Supplementary file 1 [file mmi0086-0730-SD1.pdf]

Structure of WbdD; a bifunctional kinase and methyltransferase that regulates the chain length of the O antigen in *Escherichia coli* O9a

Gregor Hagelueken[#,1], Hexian Huang[#,1], Bradley R. Clarke[2], Thomas Lebl[3], Chris Whitfield[2], James H. Naismith[1,*]

[1]Biomedical Sciences Research Complex, University of St Andrews, North Haugh, St Andrews, Fife, KY16 9ST, UK
[2]Department of Molecular and Cellular Biology, University of Guelph, Guelph, Ontario, N1G 2W1, Canada
[3]School of Chemistry, University of St Andrews, North Haugh, St Andrews, Fife, KY16 9ST, UK

email: naismith@st-andrews.ac.uk

Short title : Crystal structure of *E. coli* WbdD.

SUPPORTING MATERIAL
FIGURES S1 to S7

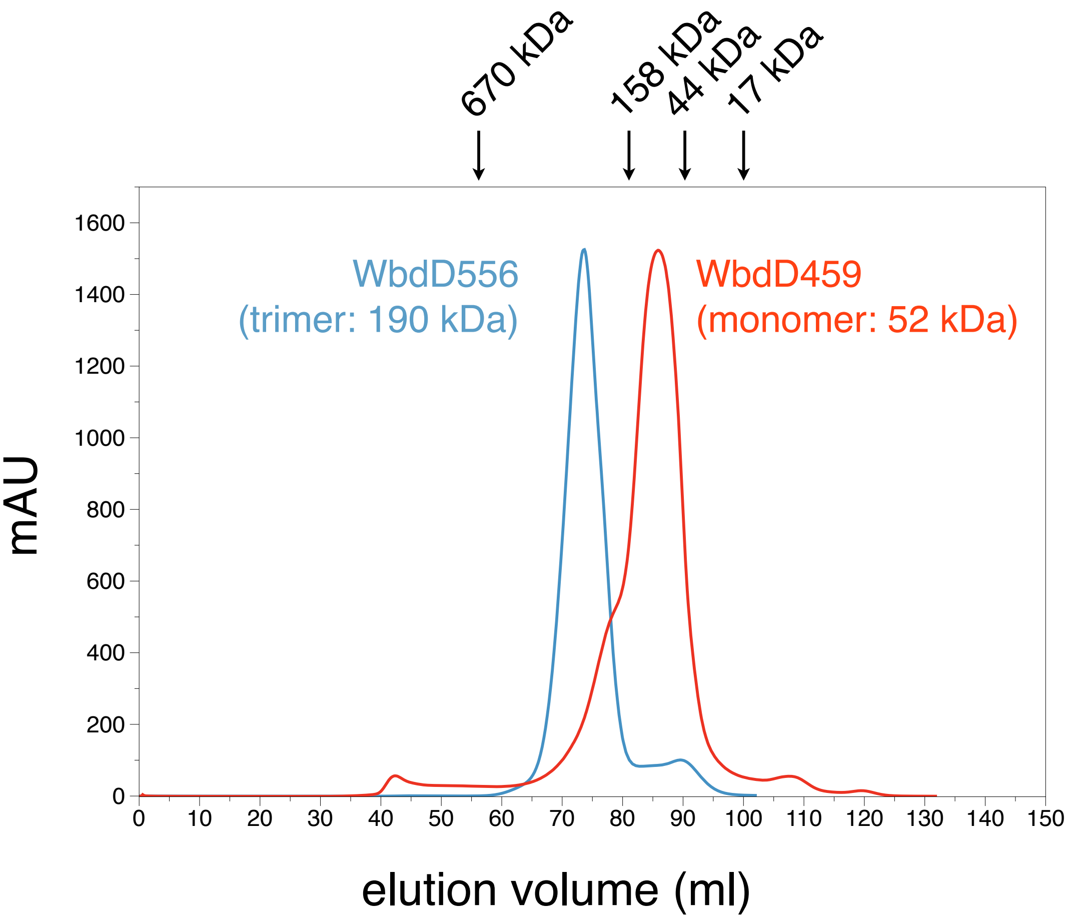

Supplementary Figure S1: Gel filtration profiles of WbdD556 (trimer, blue) and WbdD459 (monomer, red). Elution volumes of standard proteins are indicated. The experiments were run on a Superdex200 column (GE Healthcare).

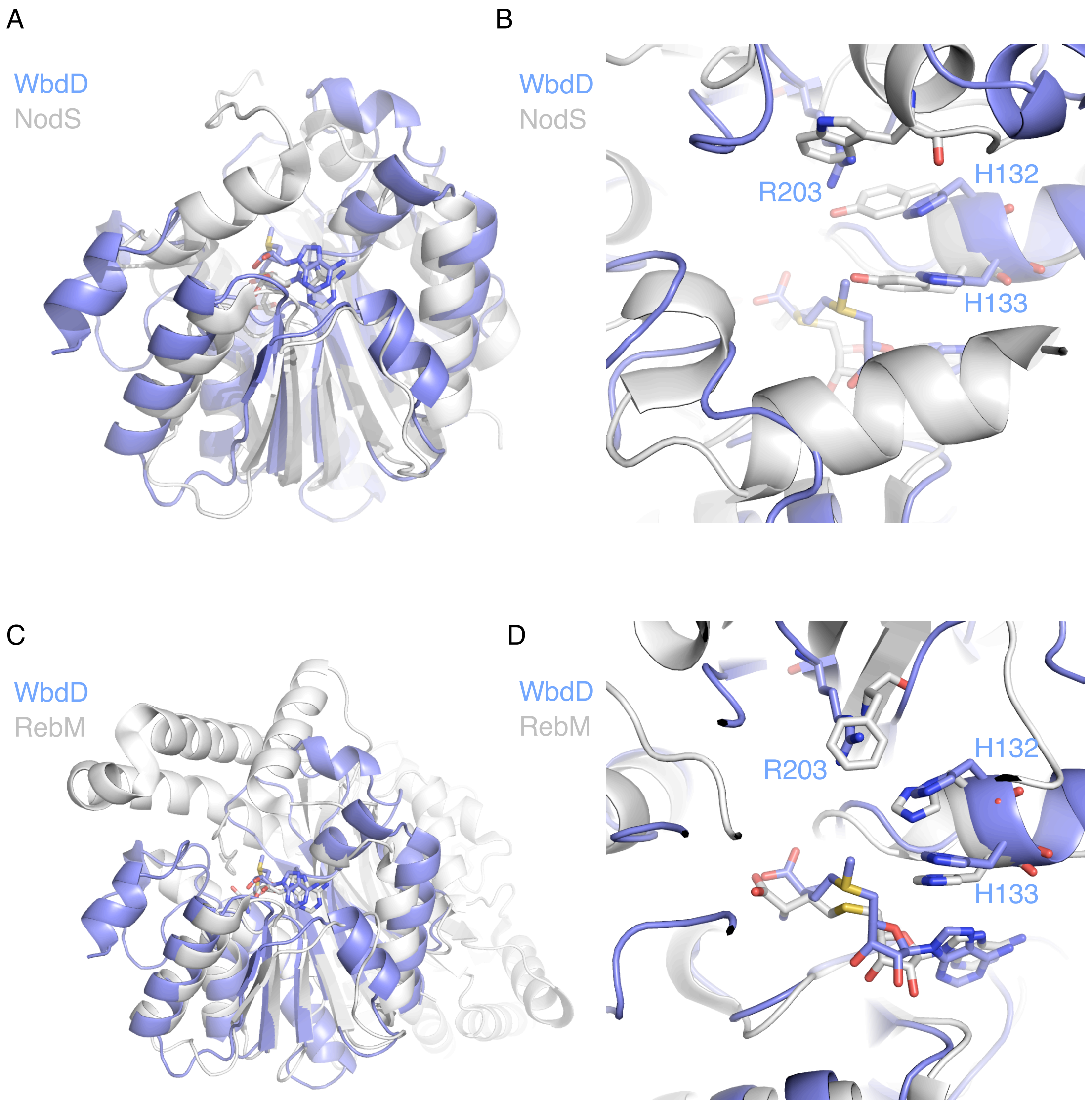

Supplementary Figure S2: The methyltransferase domain (MTase) of WbdD compared to NodS (A,B) and RebM (C,D). The proteins are shown as cartoon models (WbdD: blue, NodS/RebM: white). The SAM cofactors are shown as sticks. Selected active site residues are shown as sticks. A) and C) show the overall structures of the proteins, B) and D) are close-ups of the active site areas.

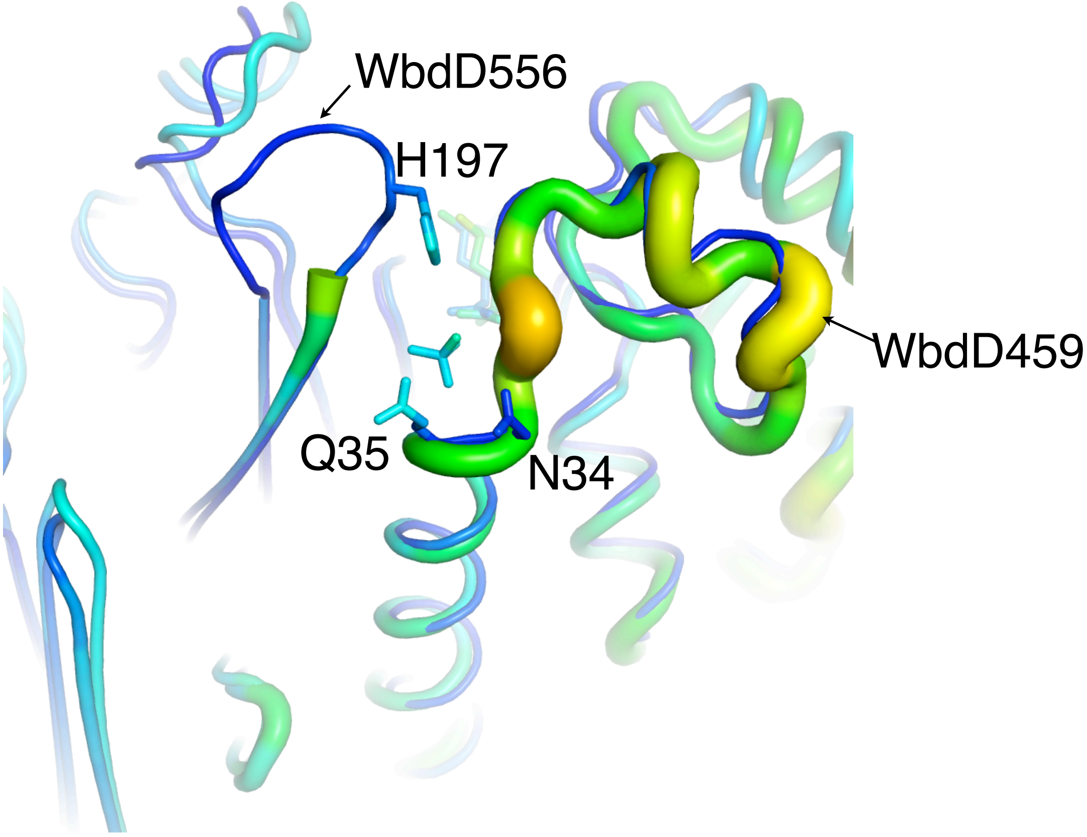

Supplementary Figure S3: The sulfate binding site is ordered by sulfate binding. The methyltransferase (MTase) domains of WbdD556 and WbdD459 are superimposed and shown in "loop" representation. The thickness of the loops represents the relative temperature factor within each structure. The color gradient ranges from blue (low temperature factor) to red (high temperature factor). The sulfate ion that is bound in WbdD556 is indicated as sticks. The SAM cofactor of both structures and the three residues that mark the entrance to the sulfate binding pocket (N34, Q35 and H197) are shown as sticks.

A

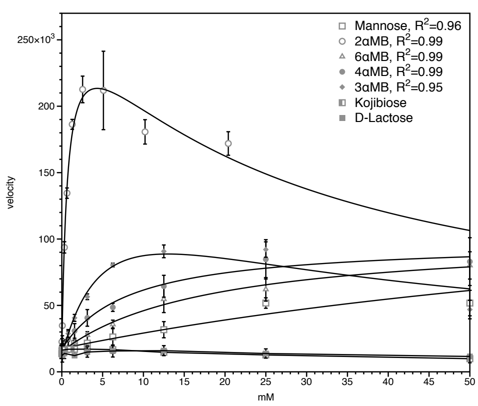

B

substrate	K_M(mM)	rel. V_{max}(%)
2αMB*	0.7±0.2	100
3αMB*	6.3±1.5	65
4αMB	8.2±1.7	41
6αMB	18.4±3.2	44
D-Mannose	>100	n.d.
D-Glucose	-	-
D-Galactose	-	-
Kojibiose	-	-
Lactose	-	-
Maltose	-	-

The kinetics showed substrate inhibition. The function "offset +Vmax(x/(Km +x*(1+x/Ki)))" was fitted to the data points by non-linear regression. The inhibitory constant Ki is 26.7 mM.

C

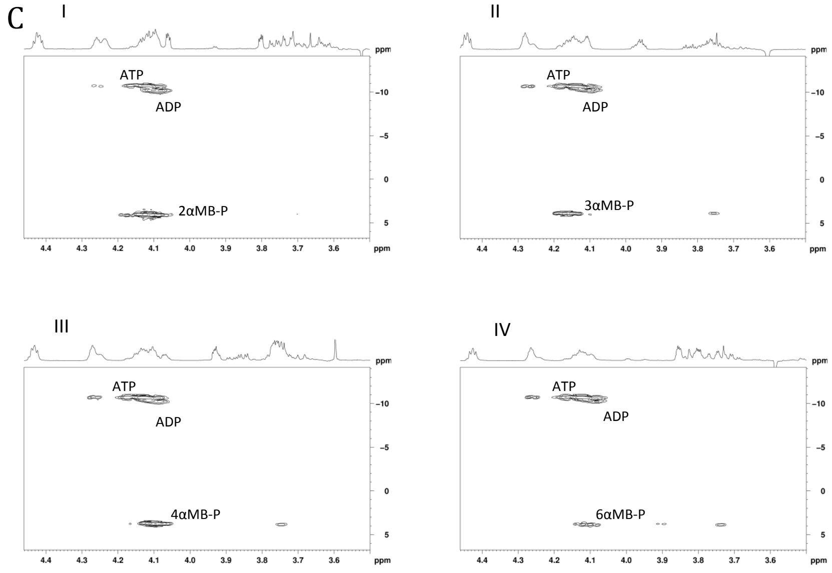

D

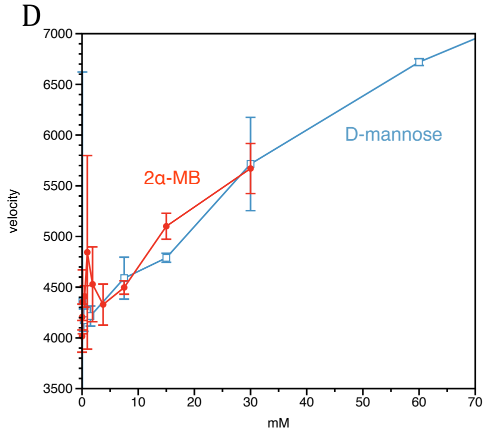

E

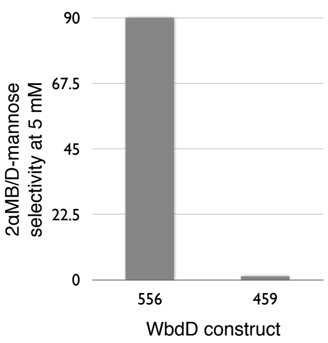

Supplementary Figure S4: A) Enzyme kinetics of the WbdD556 kinase reaction. The reaction velocity in arbitrary units (ADP glo signal, Promega) is plotted against the concentration of the indicated substrates. Data points were measured in triplicate and error bars represent the standard deviation of the measurements. Solid lines are non-linear fits to the data points using the function y=offset+Vmax*(x/(Km +x*(1+x/Ki))). **B)** Kinetic parameters and reaction velocities compared to the best substrate, 2α-MB. **C)** ^{1}H,^{31}P-HMBC spectra recorded after phosphorylation of 2α-MB **(**Panel I**)**, 3α-MB **(**Panel II**)**, 4α-MB (Panel III) and 6α-MB (Panel IV). Since cross-peaks corresponding to phosphorylated products have very similar chemical shifts in both ^{1}H and ^{31}P dimensions we assume that phosphorylation take place at the same position for all four substrates. **D)** Enzyme kinetics of the WbdD459 kinase reaction for 2α-MB (red) and D-mannose (blue). Data points were measured in triplicate and error bars represent the standard deviation of the measurements. **E)** Preference of WbdD556 and WbdD459 for 2α-MB over D-mannose. The velocites were compared at concentrations of 5 mM using the data shown in A) and D).

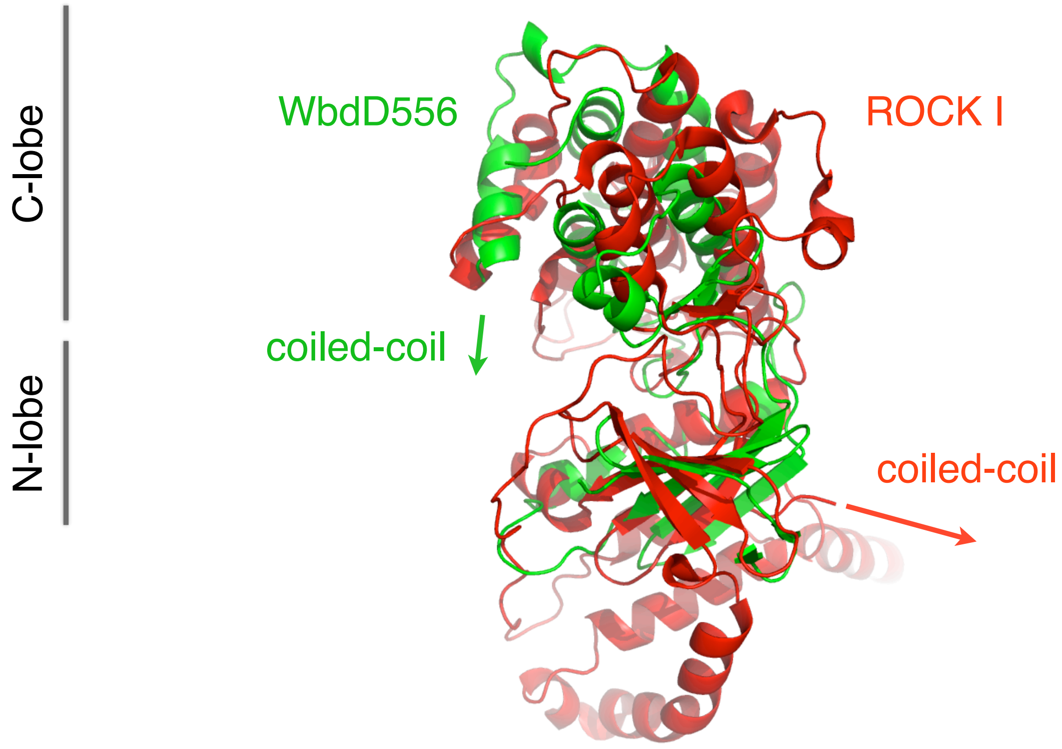

Supplementary Figure S5: Superposition of human ROCK I (Jacobs et al. (2006), red cartoon) and WbdD556 (green cartoon). The C-termini of both proteins which connect to the coiled-coil domains are indicated.

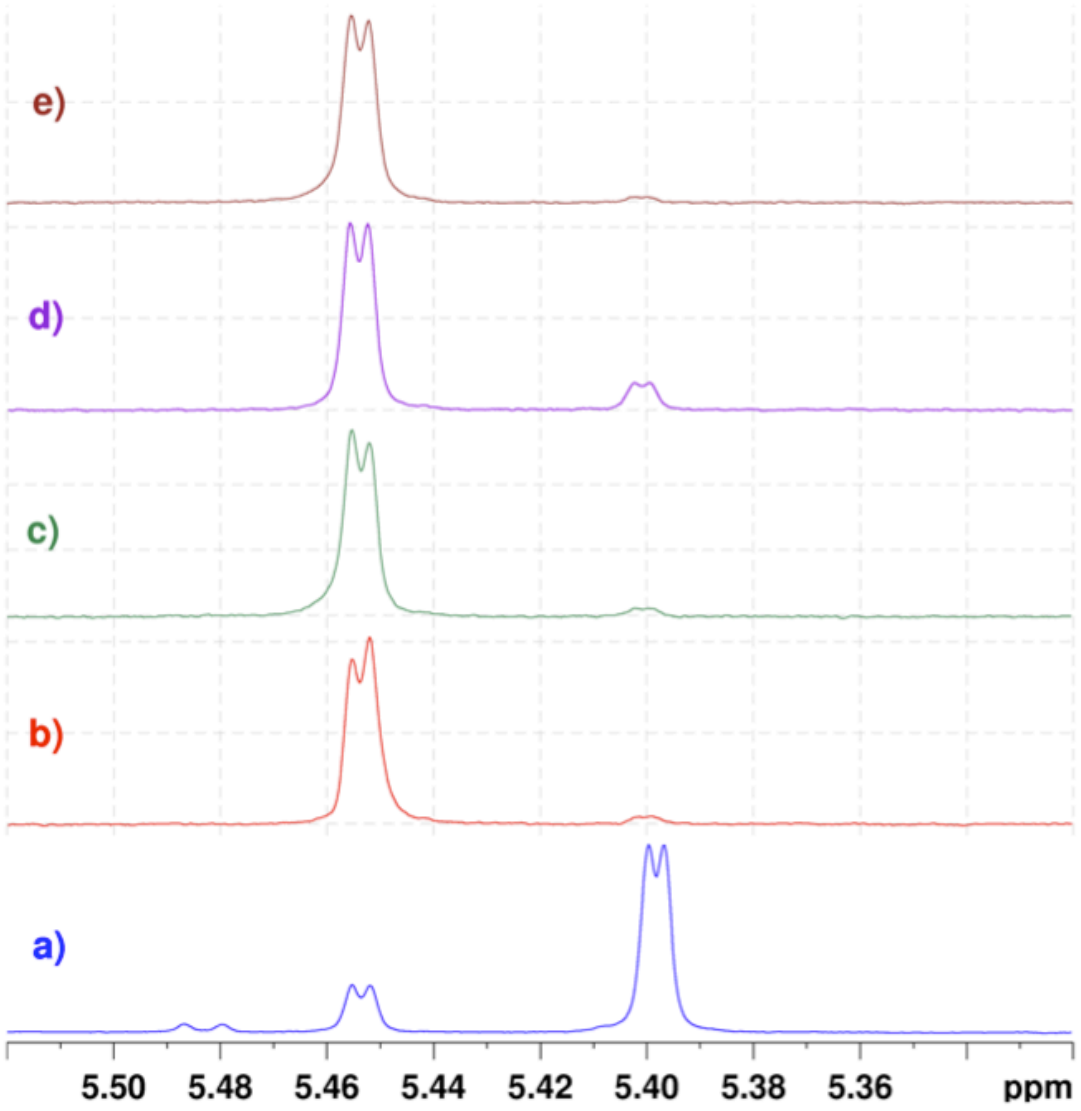

Supplementary Figure S6 a) An expansion of ^{1}H NMR spectrum showing resonances of anomeric protons of reducing sugar for 2α-MB (5.40 ppm) and phosphorylated 2α-MB (5.45 ppm) recorded during phosphorylation reaction at about 20% conversion. **b, c, d, e)** ^{1}H NMR spectra recorded prior methylation reaction with mutant proteins H132A, H133A, R203A and Y16F, respectively. One can see that conversion of phosphorylation reaction more than 85% was achieved in all four cases.

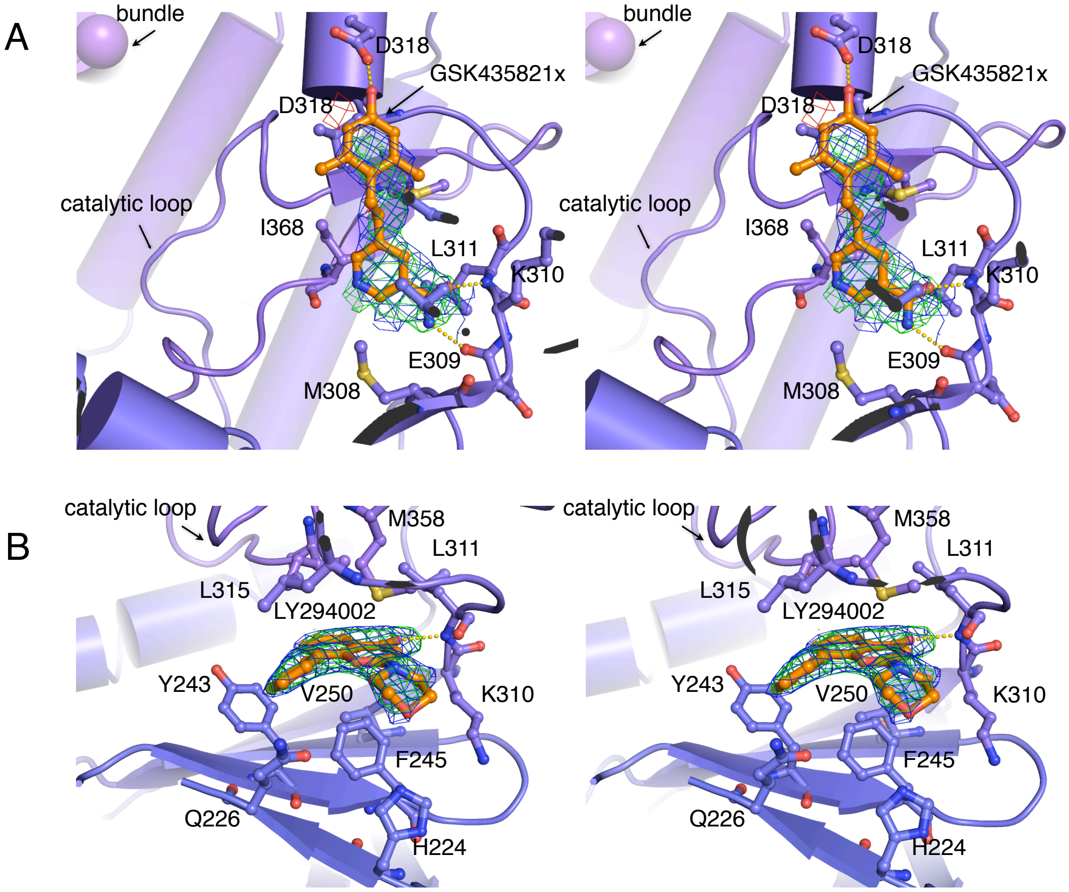

Supplementary Figure S7: Inhibition of WbdD by tyrosine kinase inhibitors. **A)** Co-crystal structure of WbdD with GW435821x (stereo pair). The inhibitor and selected residues are indicated as ball-and-stick. The blue mesh is a 2DFo-mFc omit density at 1 σ, the green mesh a DFo-mFc omit density at 3 σ. **B)** Co-crystal structure of WbdD with LY294002; Stereo pair, representation as A).
