# Supplementary material for: Structure of WbdD: a bifunctional kinase and methyltransferase that regulates the chain length of the O antigen in Escherichia coli O9a
Source: Mol Microbiol. 2012 Sep 27;86(3):730–42. doi: 10.1111/mmi.12014 (PMC3482155; doi:10.1111/mmi.12014)
Supplement: Supplementary file 2 [file mmi0086-0730-SD2.docx]

Table S1: Current structural information about eukaryotic-like kinases in bacteria and archea. To gather this information, the PDB was searched with the following parameters: “*SCOP classification*: Protein kinase-like (PK-like) (consists of two alpha+beta domains, C-terminal domain is mostly alpha helical) AND *Source Organism*: Bacteria or Archea AND removed 95% similar sequences to exclude ligand co-complexes, mutants, etc.”

| **Form of life** | **Number of unique kinase structures** | **PDB codes** | **Name** | **function** | **reference** |
| --- | --- | --- | --- | --- | --- |
| Bacteria | 6 | 2pul | MTR | 5-methylthioribose kinase | Ku et al. 2007 |
|  |  | 2ppq | ThrB | homoserine kinase | not published |
|  |  | 1zyl | YihE | Ser/Thr kinase | Zheng et al. 2007 |
|  |  | 1o6y | PknB | Ser/Thr kinase | Ortiz-Lombardia et al. 2003 |
|  |  | 1nd4, 1j7l | APH | aminoglycoside kinase | Nurizzo et al. 2003, Burk et al. 2001 |
| Archea | 1 | 1zar | Rio2 | RIO serine protein kinase | Laronde-Leblanc et al. 2005 |
